# Compensated pathogenic variants in coagulation factors VIII and IX present complex mapping between molecular impact and hemophilia severity

**DOI:** 10.1038/s41598-019-45916-3

**Published:** 2019-07-02

**Authors:** Òscar Marín, Josu Aguirre, Xavier de la Cruz

**Affiliations:** 1 Research Unit in Clinical and Translational Bioinformatics, Vall d’Hebron Institute of Research (VHIR), Universitat Autònoma de Barcelona, P/Vall d’Hebron, 119–129, 08035 Barcelona, Spain; 2 ICREA, Barcelona, Spain

**Keywords:** Molecular evolution, Protein sequence analyses

## Abstract

Compensated pathogenic deviations (CPDs) are sequence variants that are pathogenic in humans but neutral in other species. In recent years, our molecular understanding of CPDs has advanced substantially. For example, it is known that their impact on human proteins is generally milder than that of average pathogenic mutations and that their impact is suppressed in non-human carriers by compensatory mutations. However, prior studies have ignored the evolutionarily relevant relationship between molecular impact and organismal phenotype. Here, we explore this topic using CPDs from FVIII and FIX and data concerning carriers’ hemophilia severity. We find that, regardless of their molecular impact, these mutations can be associated with either mild or severe disease phenotypes. Only a weak relationship is found between protein stability changes and severity. We also characterize the population variability of hemostasis proteins, which constitute the genetic background of FVIII and FIX, using data from the 1000 Genome project. We observe that genetic background can vary substantially between individuals in terms of both the amount and nature of genetic variants. Finally, we discuss how these results highlight the need to include new terms in present models of protein evolution to explain the origin of CPDs.

## Introduction

**Figure 1:**
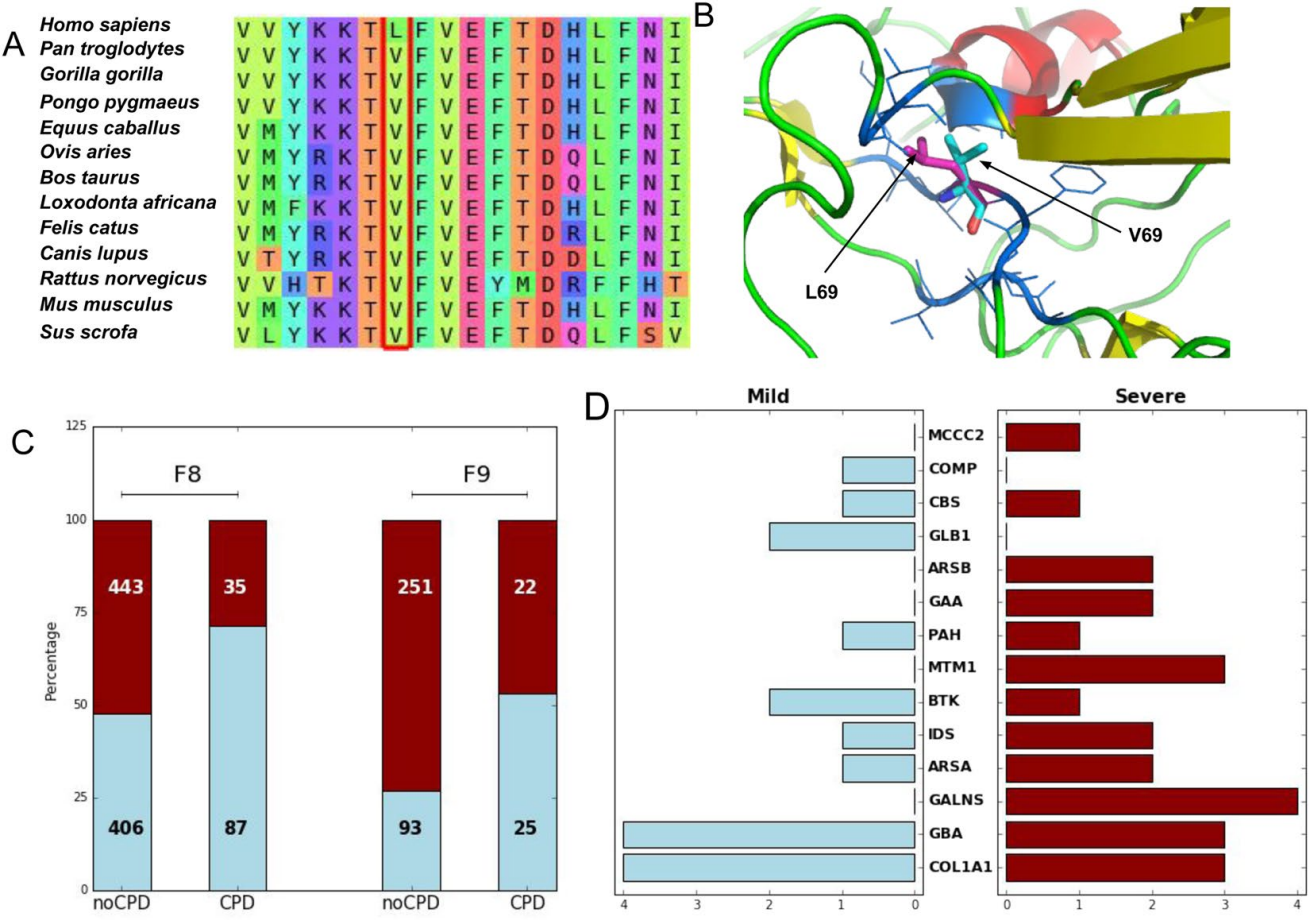
Compensated pathogenic deviations (CPDs): definition and distribution relative to disease severity. (**A**) Illustration of the concept of CPDs: a mutation that is pathogenic in humans but neutral in other species. The mutation shown in the figure, L69V, affects human FVIII and leads to hemophilia A^65^. The location of this mutation in the multiple sequence alignment of the FVIII family (in the red box) shows that valine appears to be native in other species, such as chimpanzees, mice, and rats. (**B**) Spatial neighborhood (dark blue residues) of the CPDs (native and mutant residues indicated by light blue and magenta sticks, respectively), where compensatory mutations are more likely to happen^7^. (**C**) Distribution of mild (blue) and severe (red) cases associated with CPDs and noCPDs variants for FVIII and FIX. (**D**) Distribution of mild and severe CPDs in genes for which disease severity annotations are available.

Understanding the phenotypic consequences of genetic variability is still an open challenge relevant to different areas of biology, from biomedical research^1,2^ to protein evolution studies^3–6^. A case of particular interest is that of the human sequence variants known as compensated pathogenic deviations (CPDs)^7^, which are damaging for human carriers but appear as neutral in other species (Fig. 1A). This dual aspect of the amino acid replacement reflects the two main characteristics of CPDs. First, in its human protein location, the amino acid replacement has an impact on protein structure/function big enough to cause disease. Second, in the non-human protein, this impact is modulated by a suppressor mechanism. Kondrashov *et al*.^7^ identified compensatory mutations as the main suppressor mechanism (the so-called Compensatory Hypothesis^8^) and postulated that such mutations most likely correspond to substitutions at spatial locations near CPDs (Fig. 1B). The compensatory hypothesis is strongly supported by a series of studies involving large structural analyses^9^, stability computations^8^, and comparative genomics^10^.

Within this stream of research, Ferrer-Costa *et al*.^11^ explored whether the molecular nature (e.g., protein location, changes in biophysical properties) of pathogenic deviations (PDs) determines the probability of compensation. They found that CPDs are usually less structurally disruptive than the average PDs, as they are associated with higher solvent exposure and smaller changes in physico-chemical properties. This result was confirmed by Barešić *et al*.^9^, who also found that CPDs tend to avoid residues directly involved in protein function (e.g., from binding and catalytic sites).

Together, these studies provide a good understanding of CPDs at the molecular level. However, the relationship between molecular and organismal phenotypes, which is key to the evolutionary study of these mutations, remains largely unexplored. In general, biomedical evidence suggests that there is a monotonic correspondence between the molecular impact of PDs and the clinical phenotype. For example, studies examining the development of pathogenicity predictors^2,12^ have consistently observed that small changes in molecular properties typically correspond to variants that have no impact on the individual’s health. Other studies identified a relationship between the range of the mutation effect and clinical severity. For example, Miyata *et al*.^13^ represented the impact of mutations with a continuous function of distance in the physico-chemical space. They related the values of this function to the severity of hemolytic anemia and found correspondence between the two phenotype levels. For G6PD deficiencies, Miller and Kumar^14^ found a similar trend, again using physico-chemical differences to measure the impact of a mutation. In addition, a comparable relationship between molecular properties and clinical severity was observed when the effects of a mutation were represented by experimental ΔΔG (protein stability change upon mutation)^15^ or conservation-based measures (related to the functional role of the mutated residue)^16^.

Considering that CPDs are a subset of PDs^9,11^, we expect to find a similar correspondence between molecular impact and clinical phenotype (i.e., “mild-to-mild” for most CPDs and “severe-to-severe” for the small fraction of structurally/functionally disruptive CPDs). Confirmation of this relationship would allow us to study CPDs using known models of protein evolution, which utilize measures of molecular impact as a proxy for fitness^3^. However, the situation may be more complex, as a growing amount of evidence shows that genetic background may contribute to clinical phenotype^4,17^, particularly those genes belonging to the functional module (or disease pathway) of the mutation carrier protein^18^. For example, in the case of hemophilia, it is known^19^ that genetic alterations in hemostasis proteins mitigate the clinical symptoms of the disease, and several reports relate disease severity and the effects of genetic background^20–25^.

In this work, we characterize the relationship between molecular impact and organismal phenotype in the context of CPDs. As a model system, we use the coagulation factors VIII (FVIII) and IX (FIX), two proteins whose mutations cause hemophilia A and B, respectively. Hemophilia is a well-known disease—it was first reported in Jewish writings dating to the 2nd century AD^26^—that primarily affects males. It has a characteristic bleeding phenotype^27^, the severity of which affects organismal fitness to different degrees (i.e., bleeding can be either mild or life-threatening). In the context of the present work, it is important to note two interesting aspects of research on hemophilia. First, information about disease severity is available for a large number of variants (see *Materials and Methods*) of FVIII and FIX, which will allow us to analyze the correspondence between different measures of molecular impact (at the structure and function levels) and organismal fitness, using severity as a proxy for the latter. Second, the functional module of FVIII and FIX, constituted by the proteins from the hemostasis system, has been well-described^19,28–30^. Thus, we can assess its mutational load in the general population (based on the 1000 Genomes project^31^), which is relevant for understanding the modulatory potential of genetic background. The combined results of these two analyses may help advance the evolutionary understanding of CPDs.

## Results

### CPDs in FVIII and FIX can be associated with either mild or severe forms of hemophilia

For the two coagulation factors, we found that both their CPDs and non-compensated pathogenic deviations (noCPDs) are associated to either mild or severe forms of hemophilia (Fig. 1C). The percentages are specific for each protein: for CPDs 29% (FVIII) and 47% (FIX) of the cases are associated to severe disease; for noCPDs these figures rise to 52% (FVIII) and 73% (FIX). For both coagulation factors, CPDs are less frequently associated with severe symptoms than noCPDs (Fisher’s exact test: p-value = 1.0 × 10^−6^ for FVIII and p-value = 4.0 × 10^−4^ for FIX).

We investigated other diseases in which CPDs spread over the severity scale. To do so, we used severity annotations and variant information retrieved from the UniProt database. The number of cases was small, comprising 42 CPDs (17 associated with mild disease and 25 associated with severe disease) distributed over 14 genes (Fig. 1D). For this reason, we could not draw statistically relevant conclusions for each gene. However, we found that CPDs may be associated with either mild or severe forms of disease (Fig. 1D). Treating the whole dataset as a single sample revealed no detectable differences between CPDs and noCPDs (Fisher’s exact test: p-value = 1). This result does not contradict the trends observed for FVIII and FIX since pooling data from different diseases may obscure gene-specific trends^32^.

### CPDs in FVIII and FIX tend to be mild at the molecular level

Next, we characterized the molecular impact of FVIII and FIX CPDs to determine whether they tend to be milder than noCPDs, as found in the general case^9,11^. To this end, we compared the distribution of CPDs and noCPDs for a series of properties that reflect complementary aspects of molecular impact: change in free energy upon mutation (∆∆G, used in biophysical models of protein evolution and here computed using FoldX^33^), solvent accessibility at the mutation locus in the experimental structure (a measure of the potential for structure disruption of mutations), elements of the BLOSUM62 matrix (which capture evolutionary information^34^ and can be related to the physico-chemical changes associated with amino acid replacement^35^), and conservation pattern (measured using Shannon entropy, which is related to the functional and structural role of the native residue^16^) at the mutation locus in the multiple sequence alignments of the FVIII and FIX families.

**Figure 2:**
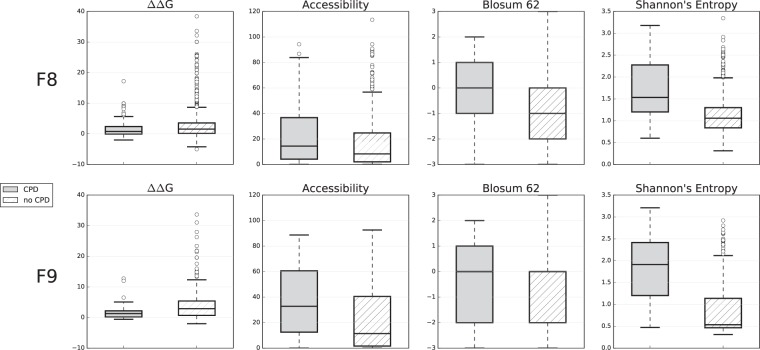
Differences between CPDs and noCPDs. For mutations of the two coagulation factors in our dataset, FVIII (top) and FIX (bottom), we computed the values of four properties: ∆∆G (change in protein stability upon mutation), relative solvent accessibility, BLOSUM62 matrix elements, and Shannon entropy. Using boxplots, we then separately represented the value distributions for the CPD (grey) and noCPD (striped) variants. There is a statistically significant tendency for noCPDs to adopt slightly more extreme values than CPDs, indicating that the latter are molecularly “milder” than the former.

We observed the same situation for both coagulation factors (Fig. 2): a significant trend for CPDs to be less disruptive than noCPDs. The p-values for the Mood’s median tests for FVIII are the same (Table 1), p-value = 0, for all the properties (∆∆G, relative solvent accessibility, BLOSUM62 elements, and Shannon entropy). The corresponding values for FIX are as follows (Table 1): 0 for ∆∆G, relative solvent accessibility, and for Shannon entropy, and 4.1 × 10^−14^ for BLOSUM62 elements. In spite of the significant differences we observed, there is an overlap between the distribution of CPDs and noCPDs in all cases, indicating that some CPDs may be as molecularly disruptive as some noCPDs. For example, if we consider ∆∆G values in the case of FVIII, the median of the CPDs distribution is above the ∆∆G value of 63% of noCPDs (Fig. 2). That is, from the perspective of free energy change upon mutation, 50% of CPDs are more disruptive than 37% of noCPDs. For FIX, the situation is similar, with 50% of CPDs being more disruptive than 29% of noCPDs.

**Table 1:** Summary of the results of the statistical tests corresponding to the comparisons shown in the different figures.

Figure	Test	p-value
Fig. 1C, FVIII	Fisher’s exact	1.0 × 10^−6^
Fig. 1C, FIX	Fisher’s exact	4.0 × 10^−4^
Fig. 1D	Fisher’s exact	1
Fig. 2, FVIII, ∆∆G	Mood’s median	0
Fig. 2, FVIII, Acces.	Mood’s median	0
Fig. 2, FVIII, Bl62	Mood’s median	0
Fig. 2, FVIII, Shan. Entr.	Mood’s median	0
Fig. 2, FIX, ∆∆G	Mood’s median	0
Fig. 2, FIX, Acces.	Mood’s median	0
Fig. 2, FIX, Bl62	Mood’s median	4.1 × 10^−14^
Fig. 2, FIX, Shan. Entr.	Mood’s median	0
Fig. 3, FVIII, ∆∆G	Mood’s median	0
Fig. 3, FVIII, Bl62	Mood’s median	2.8 × 10^−4^
Fig. 3, FVIII, Shan. Entr.	Mood’s median	0.01
Suppl. Figure 1, FIX, ∆∆G	Mood’s median	0.54
Suppl. Figure 1, FIX, Bl62	Mood’s median	0.94
Suppl. Figure 1, FIX, Shan. Entr.	Mood’s median	0.60

### The molecular impact of CPDs in FVIII (and FIX) is not strongly related to disease severity

The overlap between the distributions of CPDs and noCPDs in Fig. 2 suggests that CPDs associated to severe forms of disease (Fig. 1C) could correspond to highly disruptive mutations. To determine the extent to which this was true, we explored whether our data support a correspondence between molecular impact and disease severity. To this end, we split the CPD populations into two groups: those leading to mild and severe forms of hemophilia. We then compared these two groups in terms of the molecular-level properties examined before.

Splitting the original CPD datasets involves a reduction of the initial sample, making any ensuing comparison more sensitive to causality assignment errors (see *Materials and Methods*)^36^. To minimize this effect, we worked with a subset of the original CPD datasets with high-quality causality annotations (see *Materials and Methods*). For FVIII, the CPD sample went from 122 to 91 cases and for FIX it went from 47 to 25 cases. Then, for the comparisons in this section, these datasets were partitioned into two groups: that of CPDs associated to mild and severe disease. In the case of FVIII, the corresponding groups had 62 and 29 CPDs, respectively, and in the case of FIX, they had 12 and 13 CPDs, respectively.

**Figure 3:**
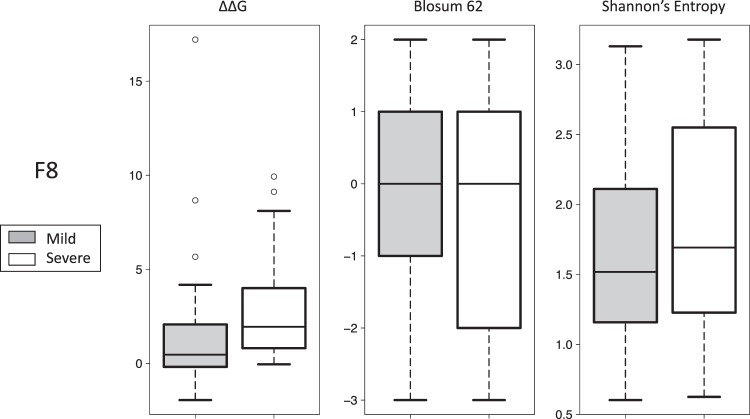
The molecular impact of CPDs and clinical severity. For FVIII, we plotted the value distribution of three properties (BLOSUM62 matrix elements, Shannon entropy, and ∆∆G) for the severe (black) and mild (grey) subsets. For ∆∆G, the difference between the distributions is statistically significant.

Comparison of FVIII CPDs leading to mild and severe disease (Fig. 3) produces statistically significant results for all the properties (Mood’s median test, Table 1): ∆∆G (p-value = 0), Shannon entropy (p-value = 0.01), and BLOSUM62 elements (p-value = 2.8 × 10^−4^). However, visual inspection of the results (Fig. 3) shows different degrees of overlap between the mild and severe distributions, consistent with deviations from a milt-to-mild/severe-to-severe relationship between molecular impact and severity phenotype. The result for ∆∆G, for which the distribution overlap is moderate, suggests that the relationship may be valid for extreme values of ∆∆G.

For the Shannon entropy, the difference between medians is surprising from a functional point of view of conservation, because the distribution for severe phenotypes is shifted towards non-conserved locations. However, the difference is small (<0.25), particularly when we consider the substantial overlap between entropy distributions (Fig. 3). In our case, the difference between medians may reflect aspects specific to the compensation of highly disruptive variants. These variants, frequent among the CPDs associated to severe disease (∆∆G plot in Fig. 3), are usually harder to compensate^11^. For this reason, we expect to find them in 3D environments where sequence changes are numerous and provide better chances of compensation^9^. In these environments, the loci of both the CPD and its neighbors will have larger entropies, and this may be reflected in the median shift described.

For FIX (Supplementary Fig. S1) the trends are similar to those of FVIII, with median differences in the same directions and large overlaps between distributions. In this case, none of the comparisons were statistically significant (Table 1) suggesting, together with the visual analysis, the presence of deviations from the monotonic relationship between molecular impact and severity. These results must be considered with care given the small sample size for FIX.

### Genetic variability in hemostasis proteins

In parallel with the previous analyses, we characterized the inter-individual variability in hemostasis proteins because evidence from biomedical^19^ studies shows that genetic alterations in these proteins can modulate the bleeding phenotype of hemophilia. To this end, we mapped the variants carried by 1233 males (obtained from the 1000 Genomes project^31^) to a set of known hemostasis proteins^30^ (19 cases that include FVIII and FIX, Supplementary Table S3). We then analyzed the resulting data in terms of amount and nature (i.e., pathogenic or neutral) of missense variants, two measures of the genetic alterations of the disease pathway related to disease severity^20–25^.

**Figure 4:**
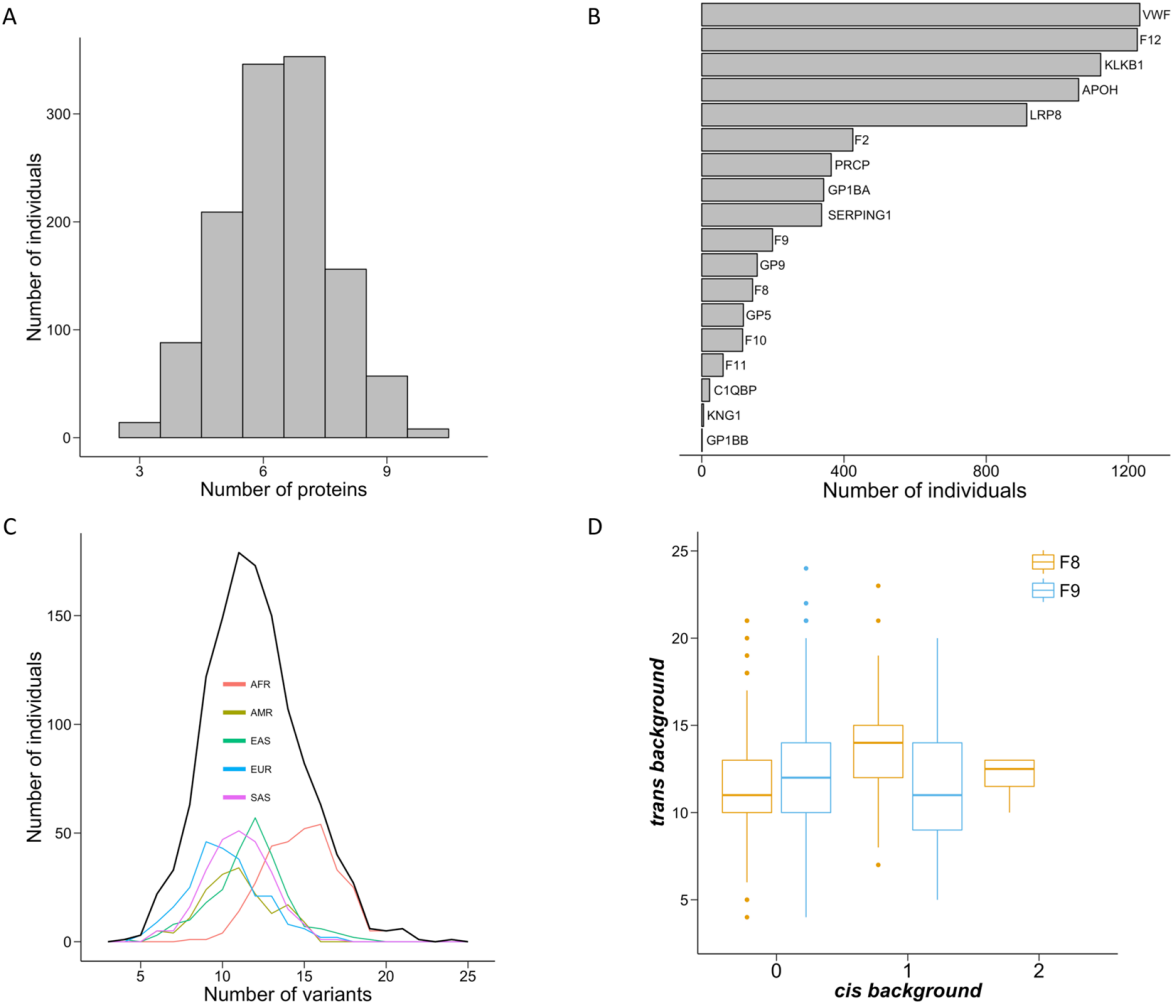
Variability in the genetic background of FVIII and FIX. This figure shows four concrete aspects of how the sequence variants from the 1000 Genomes project are distributed across hemostasis proteins (i.e. the genetic background of FVIII and FIX). (**A**) Frequency histogram of the number of mutated proteins per individual. (**B**) Number of individuals for which each hemostasis protein appears to be mutated. (**C**) Frequency histogram of the number of variants per individual. The results for the whole population are shown in black, and separate results for each of the five super-populations in the 1000 Genomes project—African (AFR), Admixed American (AMR), East Asian (EAS), European (EUR), and South Asian (SAS)—are shown in color. (**D**) Distribution of the number of variants of background proteins relative to FVIII (orange) and FIX (blue).

As Fig. 4A shows, all individuals in the population present variants in at least three of the proteins, and most frequently (in 699 of 1233 cases), individuals had variants in 6–7 proteins. However, not all the proteins are equally mutated; the von Willebrand factor (vWF) and coagulation factor FXII (F12) were mutated in almost all individuals (Fig. 4B), while variants in Kininogen-1 (KNG1) and Platelet glycoprotein Ib beta chain (GP1BB) were seldom observed.

The number of variants changes between individuals (Fig. 4C), mainly ranging from 5–20, and it is affected by ethnicity. Plotting the number of variants in hemostasis proteins (excluding those of FVIII and FIX) relative to those in FVIII and FIX (Fig. 4D) indicates different possibilities for variability in genetic background. This variability, following the notation in Jordan *et al*.^10^ (*cis*: in the same protein, *trans*: in a different protein), sometimes may be completely *trans* relative to either FVIII or FIX, and sometimes it may be a mixture of *cis* and *trans* variants. The latter is relevant because *cis* locations are believed to host compensatory variants^7^ more frequently than *trans* locations.

**Figure 5:**
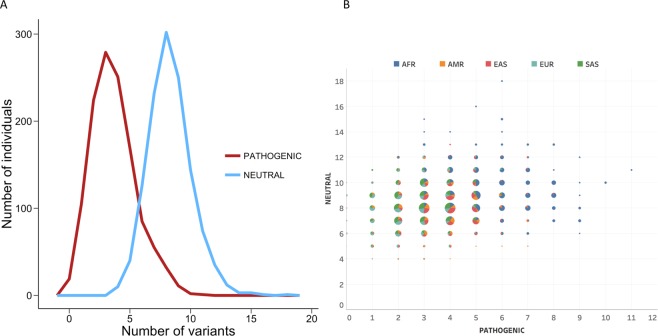
Pathogenic load of hemostasis proteins. (**A**) Distribution of the number of neutral (blue) and pathogenic (red) variants per individual in the 1000 Genomes population. (**B**) Scatterplot showing the different combinations of neutral and pathogenic variants found in the population. The size of the circles represents the number of individuals in which each combination was observed. In addition, each circle is a pie plot that represents the fraction of individuals from the different superpopulations in the 1000 Genomes: African (AFR), Admixed American (AMR), East Asian (EAS), European (EUR), and South Asian (SAS).

At the compositional level, we distinguished between neutral and pathogenic variants and looked for inter-individual differences in the number of each. For all the identified variants, we queried the HGMD^37^ database to retrieve all available pathogenicity annotations. Figure 5A shows that pathogenic variants are present in a majority of the population (98%), although neutral variants predominate. We refine this view in Fig. 5B, which shows that both variant types appear in different combinations and that some individuals have a higher number of pathogenic variants in hemostasis proteins than others.

## Discussion

Biophysical studies of CPDs using structural analyses and stability computations have explained their dual (i.e., pathogenic/neutral) behavior^3,8,10,11,38^. In particular, we know that compensatory mutations are the principal mechanism suppressing the harmful effects of CPDs^8,10^ and that these effects, in terms of molecular properties, tend to be milder than those of non-compensated PDs^9,11^ (Fig. 2). The biophysical approach has been extended to explain the appearance of CPDs during evolution using ∆∆G as a proxy for fitness either explicitly or implicitly^3,9,11,38^. More precisely, dePristo *et al*.^3^ proposed a formalism in which, upon mutation, fitness variations are expressed as an exponential function of the difference in ∆∆G from a reference value. This formalism is easily interpretable, and the authors illustrated its potential in the comparison of two competing hypotheses about the origin of CPDs. However, the explanatory power of the model is limited to those cases where there is a monotonic (mild-to-mild/severe-to-severe) correspondence between molecular impact and organismal fitness, and the effect of genetic background is small.

In our work, we explored the extent to which this is the case for CPDs in FVIII and FIX. Specifically, we studied (i) how measures of molecular impact (structural and functional) relate to the severity phenotype (Figs 2, 3 and Supplementary Fig. S1) and (ii) the compositional properties of genetic background (Figs 4, 5). For FVIII, ∆∆G was the molecular property showing the most noticeable difference between the mild and severe distributions (Fig. 3). For FIX, the trend is comparable (Supplementary Fig. S1) but statistically non-significant. This result suggests that, at least for FVIII, fitness models based on ∆∆G^3,39^ may be useful for the evolutionary study of CPDs. However, the applicability range of these models may be restricted when working with ∆∆G estimates because of their moderate correlation with observed stability changes. For example, in the case of FoldX^40^ the authors cite a value of 0.8 (r^2^ = 0.64); for the same program, Tian *et al*.^41^ find a correlation of 0.5 and a low accuracy (69.5%) for the discrimination between stabilizing and destabilizing variants. On the other hand, results from the application of FoldX to the characterization of mutations causing Fabry disease indicate^42^ that extreme ∆∆G values may successfully identify pathogenic variants. On this basis, we believe that, when working with computational estimates of ∆∆G, it may be preferable to restrict the use of ∆∆G-based fitness models to those CPDs with a large effect on stability.

For CPDs having a small effect on stability, the applicability of ∆∆G-based models is more limited. This may occur for two reasons, apart from the previously discussed problems that arise when working with ∆∆G estimates. The first reason is a low correlation between ∆∆G and protein function^43,44^; a CPD may have a small impact on ∆∆G but a large impact on protein function, resulting in a noticeable effect on fitness. However, models only based on ∆∆G would predict a minor effect on fitness. The second reason may be the modulatory effect of genetic background. Evidence from both experimental and theoretical bioinformatics studies shows that the phenotypic effect of mutations is modulated by genetic background^4,45–48^. For example, by performing RNAi experiments with two *C. elegans* isolates, Vu *et al*.^47^ found that about 20% of the ~1400 genes they tested displayed background-dependent differences in the severity of the loss-of-function phenotype. An array of biomedical studies also support the regulatory role of background^20–25^. For example, To-Figueras *et al*.^49^ found that in congenital erythropoietic porphyria, a disease caused by mutations in *UROS* (an enzyme of the erythroid heme biosynthesis pathway), severity depends on the variants present in *ALAS2*, the rate-controlling enzyme of this pathway. In the case of hemophilia, we know that genetic background must be considered because specific variants in hemostasis proteins other than FVIII and FIX can modify the severity phenotype^19^. Within this context, one expects that the cumulative effect of background variants on fitness may sometimes surpass that of CPDs with a small effect on stability, thus limiting the applicability of ∆∆G-based models in this case.

**Figure 6:**
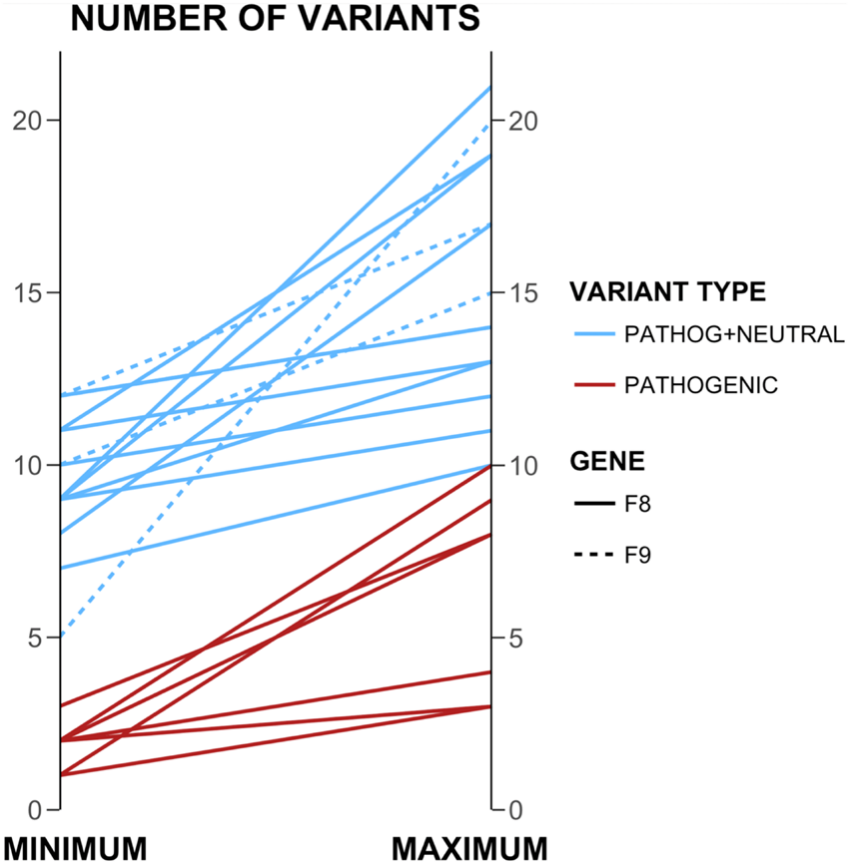
Differences in the background of specific variants between FVIII and FIX. The genetic background of a given variant can vary between individuals. Here, we focus on variants of FVIII and FIX and define background as the number of accompanying mutations in the hemostasis proteins using population data from the 1000 Genomes project. Each line represents a variant in these coagulation factors (continuous and broken lines for FVIII and FIX, respectively) that is present in more than one individual. The line unites the minimum (left axis) and maximum (right axis) number of background mutations observed for that variant. Blue indicates that all the background mutations were counted, regardless of their nature, and red indicates that only pathogenic background variants were counted. For the latter investigation, we found only examples relating to FVIII.

In the previously cited biomedical studies, 1–3 (usually pathogenic) variants in the genes of the disease pathway are enough to modulate the effect of the causal variant. In our case, after characterizing the number and kinds of variants in hemostasis proteins, we found that many individuals already carry 5–20 variants (Fig. 4C) and, frequently, one or more of these variants are pathogenic (Fig. 5, *Materials and Methods*). Another interesting aspect of our results (Figs 4, 5) is the diversity they reveal; neither background size nor composition are constant in the population (due to ethnic diversity and inter-individual variability). Comparable results are observed at the variant level; the same variant may appear with different backgrounds in different individuals (Fig. 6). Given their impact on protein stability^50^ and protein–protein interactions^51^, we expect that the coincidence of several pathogenic variants in the same individual will have a net lowering effect on the efficacy of the hemostasis mechanism. This effect will change between individuals since the number of pathological mutations varies between individuals (Fig. 5) and because the net effect of the variants may not follow a simple additive model^52^. In summary, the genetic background of FVIII and FIX has the potential to modulate the impact of CPDs.

Our results are specific for FVIII and FIX; for these coagulation factors, they suggest that, from an evolutionary point of view, we need to expand our models for the appearance of CPDs during evolution. Present models^3^ may work only for the most disruptive variants (involving large ∆∆G values or affecting functional sites); including the contribution of genetic background (e.g., applying the approach proposed for complex epistatic effects^5,53^ under a biophysical framework^38^) may be relevant when the mutations under study have a mild impact on protein function. Extension of this conclusion to other proteins will require additional work showing, among other things, that molecular factors related to protein function (e.g., protein interactions, complexity of the functional module, etc.) support a modulatory role for genetic background. We believe, however, that the modification of fitness models to take into account the effect of background must be accompanied by an effort to increase the accuracy of ∆∆G computations. Otherwise, increasing the complexity of the models will augment the error of fitness estimates.

## Materials and Methods

### CPD dataset

To obtain our sets of CPDs for FVIII and FIX, we followed a three-step protocol. First, for both coagulation factors, missense pathogenic mutations were retrieved from the databases CHAMP for Hemophilia A (http://www.cdc.gov/ncbddd/hemophilia/champs.html)^54^ and CHBMP (http://www.cdc.gov/ncbddd/hemophilia/chbmps.html)^55^. The pathogenicity of these variants was validated using ClinVar^56^. We identified two and three cases for FVIII and FIX, respectively, that were considered benign according to Clinvar^56^. These were removed from our dataset. Thus, we obtained a total of 971 mutations for FVIII and 391 for FIX. We annotated these mutations with the severity phenotype provided in the “Reported Severity” field in the CHAMP/CHBMP databases, which indicates the phenotypic presentation of the disease (e.g., bleeding patterns). Second, we built a multiple sequence alignment (MSA) for the two coagulation factors, as described below. Third, for each mutation, we checked if at the MSA location of the wild-type residue we could find the mutant residue in another species (Fig. 1A). When this was the case, the mutation was considered a CPD. At the end of this process, we had obtained 122 (87 mild, 35 severe) and 47 (25 mild, 22 severe) CPDs for FVIII and FIX, respectively. In some analyses (Fig. 2), we used noCPDs. We obtained 849 (406 mild, 443 severe) and 344 (93 mild, 251 severe) noCPDs for FVIII and FIX, respectively. The complete list of mild/severe mutations used in this work is provided in Supplementary Table S1.

In the Results section “The molecular impact of CPDs in FVIII …” we compare the molecular properties of CPDs (∆∆G, Blosum62 matrix elements and Shannon Entropy) associated to mild and severe versions of the disease. For this comparison we had to partition the set of CPDs into two subsets, corresponding to the variants associated to mild and severe versions of the disease, respectively. The size of these subsets was relatively small (87 mild and 35 severe for FVIII; 25 and 22 mild and severe for FIX) making the comparisons more sensitive to experimental error^36^, which in our case corresponds to the uncertainty level in the causality assignment of the variants. To reduce this effect to a minimum, we manually verified the causality annotations of each CPD, using the references provided in the CHAMP/CHBMP databases. In particular, we checked to which extent the criteria employed to establish causality were comparable to the most recent recommendations in the field^57^: we looked for evidence^57,58^ such as uniqueness of the variant in the carrier’s sequence, use of healthy individuals as controls, and structural/functional analysis of the variant’s impact and conservation at the mutation locus. We discarded those CPDs for which the evidence of causality was unclear (that is, it was either not mentioned or appeared to be weak). At the end of this process, the final number of CPDs was: 91 (62/29 corresponding to mild/severe disease) for FVIII and 25 (12/13 corresponding to mild/severe) for FIX. Given the small sample size of the FIX dataset, we decided to limit the comparisons in Fig. 3 to FVIII and present the results for FIX in Supplementary Fig. S1. The final sets of manually curated CPDs are provided in Supplementary Table S2.

For the remaining proteins (Fig. 1D), CPDs were obtained as follows. First, we queried the UniProt^59^ database with the keywords “lethal/severe” and “mild”. Of the resulting set of proteins, we kept only those for which there were at least five instances of each case. We then followed the second and third steps of the protocol for FVIII and FIX (described in the previous paragraph): we constructed an MSA for each protein and examined the MSA columns of the human native residues to determine whether there were pathogenic residues in the non-human species. At the end of this process, we had retrieved 155 mild and 229 severe mutations that led to 17 mild and 25 severe CPDs distributed over 14 proteins (Fig. 1D and Supplementary Table S1). In some analyses, we used noCPDs, of which there were 138 and 204 mild and severe cases, respectively.

The final list of mutations is provided in Supplementary Table S1.

### Characterization of mutations in terms of molecular properties

In this study, the molecular impact of mutations is described using four parameters: protein stability change upon mutation (∆∆G), solvent accessibility, elements of the BLOSUM62 matrix, and Shannon entropy at the mutation locus. These parameters, or related ones, are routinely used to characterize pathogenic mutations and reflect different aspects of their impact on protein structure and function^2^. In particular, ∆∆G is a central parameter in the biophysical theory of protein evolution^3,38^, and it was recently used by Xu and Zhang^8^ to test the compensation hypothesis. We estimated ∆∆G using the FoldX suite^33^. Relative solvent accessibility (obtained from the experimental structures of FVIII—PDB code 2R7E—and FIX—PDB codes 1CFH, 1IXA, and 3LC5), which indicates whether a mutation may be structurally disruptive or affect protein–protein interactions^12^, was computed with the NACCESS program^60^. BLOSUM62 matrix elements, obtained by Henikoff and Henikoff^61^ from the frequency of amino acid exchanges in blocks of aligned sequences from conserved protein regions, capture some aspects of molecular evolution^34^. It has been shown^35^ that BLOSUM matrices summarize the changes in physico-chemical properties (hydrophobicity, size, charge) associated with amino acid substitutions and related to changes in protein function and structure. This parameter was employed, among others, by Ferrer-Costa *et al*.^11^ to show that CPDs are milder than PDs. Finally, Shannon entropy at the mutation locus in the MSA is a measure of the conservation pattern at this position in the MSA of the protein family^62^, which is related to functional/structural restraints. It is equal to −Σ_i_p_i_.log(p_i_), where i runs over all the amino acids at the mutation’s MSA column. Shannon entropy varies between 0 and 4.322, with low and high values indicating highly and poorly conserved locations, respectively.

### Multiple sequence alignments

For each protein in our dataset, we built a corresponding MSA by (i) retrieving from Ensembl^63^ the mammalian orthologs of the human protein and (ii) aligning them with the program Muscle^64^.

### Hemostasis proteins

The primary biological roles of FVIII and FIX are to contribute to hemostasis^29^. This defensive mechanism is responsible for minimizing the blood loss resulting from vascular injury through the coordinated action of several proteins^29^. Both biomedical/clinical^19^ and evolutionary^30^ studies have shown that variants in these proteins can modify the bleeding patterns of carriers, a key phenotype of hemophilia. On this basis, for the genetic background of FVIII and FIX, we used a list of 19 proteins (17 cases plus FVIII and FIX; Supplementary Table S3) that was recently compiled^30^ to study the genomic basis of phenotypic variation in hemostasis.

### Variants in the 1000 Genomes project

In the last section of this study, we examined the amount and composition of the sequence variants present in hemostasis proteins in a population of healthy individuals. We obtained the relevant data from the 1000 Genomes project^31^. Specifically, we retrieved all the missense variants in the 19 hemostasis proteins listed in Supplementary Table S3 carried by each male in the 1000 Genomes database.

## Supplementary information

Supplementary Figure S1

Table S1

Table S2

Table S3

## Data Availability

All data generated or analyzed during this study are included in this article (and its Supplementary Information).
