# Supplementary figures and images for: Compensated pathogenic variants in coagulation factors VIII and IX present complex mapping between molecular impact and hemophilia severity
Source: Sci Rep. 2019 Jul 2;9:9538. doi: 10.1038/s41598-019-45916-3 (PMC6606640; doi:10.1038/s41598-019-45916-3)

F9

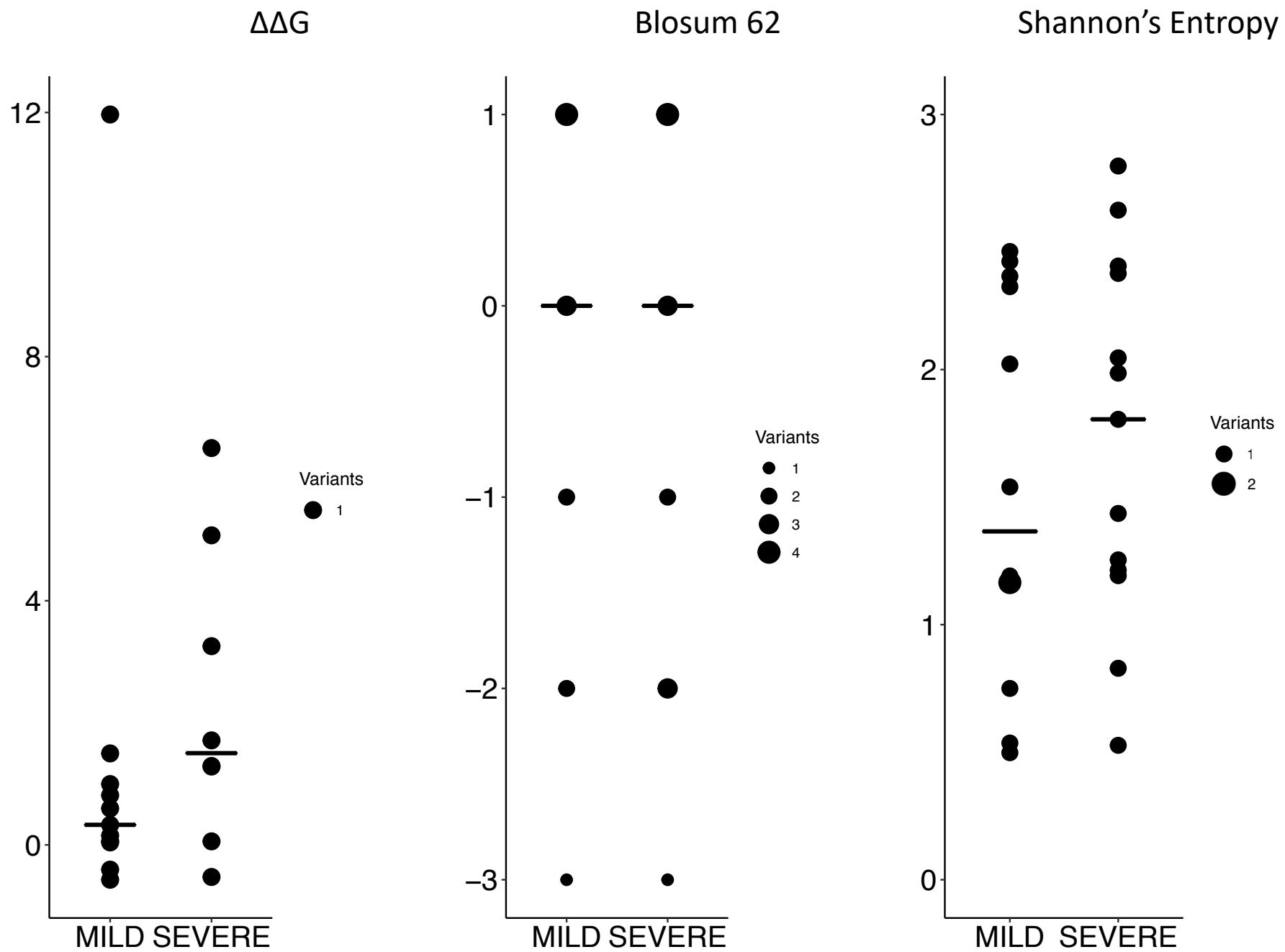

Supplement: Supplementary file 1 — Supplementary Figure S1 [file 41598_2019_45916_MOESM1_ESM.pdf]
